# Why don't politicians talk about meat? The political psychology of human-animal relations in elections

**DOI:** 10.3389/fpsyg.2023.1021013

**Published:** 2023-06-23

**Authors:** Sparsha Saha

**Affiliations:** Department of Government, Harvard University, Cambridge, MA, United States

**Keywords:** political behavior, meat, political psychology, voters, animal rights, politicians, social norms, leaders

## Abstract

Building on literature from political science and psychology, I argue that political attention on animals and animal-friendly political candidates cause voter backlash. I test this using two different kinds of experiments with large, representative samples. I ask respondents to consider political candidates running for office in a U.S. presidential primary context. I find that, overall, political attention on the need to reduce meat consumption for environmental reasons caused voter backlash compared to both a control condition and attention on the need to reduce reliance on gasoline-powered vehicles (also for environmental reasons). But, the heterogeneous effects of partisan identification were strong: voter backlash was mainly driven by Republicans and Democrats were neutral. Surprisingly, candidates who put attention on farm animal rights during elections faced no voter backlash from Republicans or Democrats. Animal-friendly candidates, particularly Black women and Latinas, with attributes that demonstrate personal concern for farm animals and strong support for animal rights generally fared very well in elections, receiving large boosts in voter support. This work launches a research agenda in political psychology that “brings the animal in” to politics.

## Introduction

Most scientists now agree that our climate change goals will not be met without addressing food, particularly animal products (Weathers and Hermanns, 2017; Shukla et al., 2019; Clark et al., 2020; Harwatt et al., 2020). According to the FAO, the livestock supply chain represents 11–20% of total global GHG emissions (GLEAM^1^; Poore and Nemecek, 2018; Gerber et al., 2013; Xu et al., 2021; Tubiello et al., 2022). In the United States, a dietary shift to plant-based foods has the potential to reduce food's emissions by 61–73% due to American over-consumption of meat (Poore and Nemecek, 2018). Animal-based foods also involve tremendous land-system changes: 83% of the world's farmland is used to produce meat, aquaculture, eggs, and dairy, yet these outputs provide just 18% of all calories and 37% of all protein globally produced (Poore and Nemecek, 2018). In the United States, where 41% of all land in the contiguous states is used for livestock (pasture and cropland) (Merrill and Leatherby, 2018,^2^), there is great opportunity if the diet-land-climate change nexus is recognized (Eshel et al., 2019) due to livestock's enormous carbon opportunity cost (Hayek et al., 2021). As the main driver of natural habitat loss worldwide and the largest anthropogenic land use type, the production of animal-based foods (including commercial fishing) is likely the leading cause of modern species extinctions (Steinfeld et al., 2006; Machovina et al., 2015; D́ıaz et al., 2019). In Central US (and other hotspots in China and India), the planetary boundary for freshwater use has been exceeded already *due to* livestock (Leng and Hall, 2021; see also, Richter et al., 2020; Mekonnen and Hoekstra, 2012).

But, despite the large environmental costs of animal-based foods, governments have done little if nothing to address this issue. In the U.S. context, the very opposite has been the case with growing subsidies that facilitate the production of cheap meat (Howard, 2019; Sewell, 2019), legislative restrictions that exclude factory farms from having to report their emissions and waste to the EPA (Miller and Muren, 2019), and a virtual lack of policy to address the need to reduce American over-consumption of meat. Even the progressive left, which we might expect to be the most vocal given their environmental agenda, has barely acknowledged the role that animal agriculture plays in climate change and other critical environmental areas like biodiversity loss.

To transition away from environmentally costly animal-based foods and ensure food and water sustainability in the future, scholars argue that political leaders have to play a role (Fuchs et al., 2016; Moberg et al., 2021). Yet, scientists are surprised that there is so much political reticence around meat given the dire need to address the health and environmental problems associated with producing it, “Politicians and policy makers demonstrate little, if any, interest in strategies to reduce meat consumption and to encourage more sustainable eating practices” (Dagevos and Voordouw, 2013; see also Springmann et al., 2017). What explains this lack of attention from policymakers? I test one reason that is usually assumed to be true in public and scholarly discussions of meat and politics in the U.S. context: voter backlash. The common wisdom in political science is that environmental issues are “vote losing” (Bodansky, 2007; Carter and Ockwell, 2007; Ockwell et al., 2009; Rabe and Borick, 2012), yet this might not be the case for all types of voters, particularly Democrats (Merkley and Stecula, 2018; Fiorino, 2022). To the best of the author's knowledge, this is the first work in political science to examine whether political attention on a particular environmental issue area—meat—is vote-losing by exploring voter evaluations of a hypothetical U.S. presidential primary candidate who puts this topic on his formal political agenda in an election context.

Strategies to encourage people to eat less meat include “nudges” that restructure the physical environment (Garnett et al., 2019) and direct appeals based on environmental and/or health information (Bianchi et al., 2018; Jalil et al., 2020; Wolstenholme et al., 2020), but there has been much less attention on the effectiveness of moral appeals related to animal rights (Bianchi et al., 2018). According to a recent meta-analysis, appeals to farm animal welfare did reduce meat consumption with large effects (RR = 1.22 with *p* < 0.05, Mathur et al., 2021). To the best of the author's knowledge, there is no experimental work in political science that assesses how political leaders might fare among voters if they use moral appeals about animal rights. The common wisdom from work in political theory is that animal rights is seen as electorally costly and thus avoided in the politics of Western nations because of “the perception that advocating for animal rights will end up harming the struggles of other disadvantaged groups” (Kymlicka and Donaldson, 2014, p. 118). Given the promise of moral appeals to reduce meat consumption (Palomo-Vélez et al., 2018), and the fact that, in reality, it is minoritized communities in the United States who bear the brunt of industrialized animal agriculture's environmental and health costs (Guenther et al., 2005; Nicole, 2013; Son et al., 2021), I also examine voter evaluations of hypothetical political candidates who put political attention on animal rights and who have animal-friendly attributes associated with animal rights (like veganism, for example) in a U.S. presidential primary context.

Study 1 employs a vignette experiment to investigate whether political attention on meat's environmental costs or animal rights engenders voter backlash in a presidential primary context. In Study 2, I use a conjoint experiment, which allows me to vary the race and gender of the hypothetical political candidate running in the presidential primary, to explore more fully the surprising result of the first study (i.e., no voter backlash from Democrats *or* Republicans against a candidate who puts attention on animal rights) by measuring voter evaluations of the kinds of candidates most likely to put animals on the political agenda.

## Where's the beef in U.S. politics?

Despite the significant and growing costs of animal agriculture, there has been virtually no development of policies that would reduce this sector's impact. Policy options for governmental action on this issue range from taxation to induced innovation (and many more; see Global Panel on Agriculture and Food Systems for Nutrition, 2020 for overview). But, when even low cost or “negative” cost proposals are not adopted (Wreford et al., 2017), the problem of *inattention*, not lack of action, is necessary to solve first before policymakers can begin to sort out which recommended actions should be or could be implemented.

Study 1 focuses on the electoral effects of political attention on animals. I use the phrase “political attention on animals” throughout the rest of the paper to refer to attention on two areas—meat's environmental costs and farm animal rights. Study 1 includes two treatment conditions that test the effects of attention on both of these areas, which previous research shows can lead to individuals indicating that they intend to reduce their meat consumption.

Political attention is a type of public agenda setting—the process through which political actors prioritize information, such that “attention [is] allocated to some problems rather than others” (Jones and Baumgartner, 2005, p. 8–9). This kind of political attention is distinguished from other forms of attention from policymakers, like supporting public investment in the alternative proteins sector through congressional appropriations and/or other less publicized methods (see, for e.g., The Good Food Institute's strategic plans, which highlight some of this work).^3^ Political actors can engage in agenda-setting in multiple ways (Jones and Baumgartner, 2005), and elections often serve as focal points, when media and public engagement is high (Johnson, 2013). During elections, candidates and parties convey messaging around their planned agenda through a host of platforms, including television advertising (Sweetser et al., 2008), press releases (Tedesco, 2005), party manifestos (Gabel and Huber, 2000), and speeches (Laver et al., 2003; Oliver and Rahn, 2016). Existing work in political attention relies heavily on political speeches and manifestos, in particular (Jennings and John, 2009; Hemphill and Schöpke-Gonzalez, 2020). For this reason, I use an experiment in Study 1 involving a hypothetical political candidate's stump speech as a way of cuing public political attention to measure voter reactions.

Study 2 investigates the electoral effects of various attributes commonly associated with candidates who are most likely to put political attention on animals: vegan dietary preference, strong support for animal rights, and personal concern for farm animals (in particular). Conjoint analysis, used in Study 2, allows for tests of multiple hypotheses by independently randomizing numerous candidate characteristics in a single experiment. Most relevant to this topic is the gender and race of the hypothetical political candidate. Given that the issue of animal rights is avoided by American policymakers due to a fear that taking it up might harm the interests of disadvantaged human subgroups (Kymlicka and Donaldson, 2014), and that women comprise the vast majority of animal rights activists (Gaarder, 2011), it is possible that the electoral effects of animal-friendly attributes depend on the gender and race of the political candidate, making intersectional analysis highly relevant to understanding how voters might react.

### The political basis of voter backlash

There are some obvious explanations for the political reluctance to address meat in the United States. The meat industry in the United States is a centralized and powerful political force, wielding both regulatory and legislative influence. Special interests and lobbying groups in this sector have spent millions supporting policies and candidates who are friendly to public investments in and virtually no regulation of meat that keep prices well below true environmental and social costs (Nestle, 2013; Simon, 2013; Lazarus et al., 2021). Yet, compared to other large corporations who spread lots of money around, the meat industry “targets their approach to a small number of key lawmakers and regulators that have a direct impact on their business interests” (Johnson, 2016, p. 1). This targeted approach, which has been very successful, nonetheless implies that not all politicians are constrained by the powerful meat industry in the United States.

What is surprising then is the virtual universality of this political reticence in the United States, from even those who do not receive support from the meat industry and also those who have centered the environment on their agendas on the far left. For example, amongst those who do not receive support from the meat industry are key (national-level) vegan/vegetarian politicians, including Cory Booker, Tulsi Gabbard, Jamie Raskin, and Adam Schiff. None of these politicians have ever formally included meat and/or animal rights on their political agendas during elections. On the far left, despite the long time relevance of the environment to progressive politicians, the Green New Deal does not even once directly mention animal agriculture, beef, or livestock, though it explicitly addresses vehicles, transit, power grids, rail, and manufacturing (H.Res.109, 2019). An accompanying official document to the Green New Deal did reference cows, but it was quickly redacted.

While important and revealing, economic reasons, by themselves, are insufficient to explain the totality of this lack of political attention in the United States. But, what about political explanations? The notion that bringing up this topic is vote-losing has been assumed to be true in news coverage, particularly by public opinion leaders like Bill Gates (Temple, 2021), Steven Chu (McMahon, 2019), and Michael Pollan (Pollan, 2011). My fieldwork has also confirmed this sentiment: key policymakers who might otherwise be receptive to talking about meat or farm animals are concerned about how voters might react.^4^ Historically, Rude (2016b) argues that it was the American passion for beef that caused the Democrats to lose control of Congress in 1946 (for the first time in 16 years), widely known as the “Beefsteak Election.” Even though the economy was soaring, President Truman's imposition of a price ceiling on meat led to a meat shortage that caused a public uproar: “Using their rights as free citizens, voters went to the polls in 1946 declaring *no meat — no vote*” (Rude, 2016a). More recently, Kamala Harris (Blum, 2020) and Alexandria Ocasio-Cortez (Remnick, 2019) have both been accused by Republicans of trying to “*take hamburgers away*,” even though they have avoided the topic formally. Outside of the United States, similar patterns have emerged: a Spanish politician (Burgen, 2022) and a French Mayor (Cohen, 2021) who faced backlash because of their comments that linked meat to the environmental crisis. In 2012, a fat tax (effectively a meat and dairy tax) in Denmark was repealed after just 1 year due to unprecedented public and private sector fallout (Bødker et al., 2015).

Political scientists widely view the environment as “bad politics” (Bodansky, 2007). Broadly, some scholars argue that climate policies designed to curb fossil fuel use are perceived by some voters as financially costly (Hann, 1986; Carter and Ockwell, 2007; Rabe and Borick, 2012). Through this economic lens, the implication is that there might likely be broad or diffuse public support for political attention on climate change (Aldy et al., 2012; Ansolabehere and Konisky, 2016; Carmack et al., 2022), but concentrated or strong opposition to it in the specific areas or industries where the costs are imposed, which can lead to voter backlash against incumbent governments that is spatially distributed (Stokes, 2016). To the author's knowledge, there has been no experimental testing in the candidate evaluation literature within American Political Science to examine the effects of (proposed) climate policies on voter support during elections.

Political psychologists challenge the notion that climate policy has diffuse public support. Using this lens, voter support for a political candidate who centers an environmental agenda would strongly depend on partisanship. Public attitudes toward climate politics are polarized, with more negative attitudes among Republicans than Democrats (Van Boven et al., 2018; Fiorino, 2022). In the United States, compared to Republicans, Democrats show higher levels of knowledge about the environment (Stoutenborough and Vedlitz, 2014) and are generally more receptive to scientific messaging around climate change (Gauchat, 2012; MacInnis and Krosnick, 2020). The main psychological mechanism to explain climate change attitude polarization based on political identification is motivated reasoning (Bayes and Druckman, 2021), which involves people using information that confirms their existing beliefs (related to their partisan identity) and rejecting that which contradicts them (Clayton and Manning, 2018). For Republican voters, skepticism about the existence or scale of climate change, and (more generally) lower pro-environmental beliefs and attitudes (Gifford and Nilsson, 2014), is explained by a higher anthropocentric worldview (Fortuna et al., 2021), as well as exacerbating top-down influences of conservative and well-funded political elites on their climate opinions (Skocpol and Hertel-Fernandez, 2016; Hahnel et al., 2020).

The transportation sector in the United States provides evidence toward the spatially distributed nature of voter support for political attention on fossil fuels, as well as heterogeneous effects on it based on partisanship. Considerable political attention on the negative impacts of traditional modes of transportation like cars on climate change has led to meaningful policy changes and greater public funding for developing and scaling up alternatives (Meckling and Nahm, 2018). But, while there could be broad or general voter support for a climate agenda that tackles transportation's role in climate change, there is certainly voter backlash in places where the costs of this fossil fuel phase-out are concentrated (Egli et al., 2022). In addition, public support for phasing out fossil fuel cars depends on party identification: the vast majority of Democrats (~70%) support the implementation of phase-out policies in 2020, but less than 50% of Republicans do so (Rinscheid et al., 2020). Using a social identity framework, Sintov et al. (2020) argue that significantly lower electric vehicle (EV) adoption among Republicans is due to their weaker symbolic attribute perceptions, or the extent to which EVs reinforce aspects of their self-identity as *Republican*.

However, there is a dearth of any work in the various fields of political science on the environmental politics of meat, in particular. Meat presents a potential challenge to the distributional analysis conducted by political economists, making it unlikely that there would be broad voter support for political attention on the environmental costs of meat, because the vast majority of Americans over-consume large quantities of animal-based foods (Willett et al., 2019) and exhibit high levels of attachment to the symbolic value of meat in their social and cultural lives (Heinz and Lee, 1998; Nguyen and Platow, 2021). In addition, Americans exhibit low levels of knowledge about the environmental impacts of animal-based foods (Camilleri et al., 2019). Still, it is likely that voters in rural areas, where factory farms and slaughterhouses tend to be located, may be most opposed to putting meat on the political agenda because it is in these areas where the costs of lower meat consumption would be concentrated.

The work on climate politics and fossil fuels in political psychology suggests that voter support for political attention on meat's environmental impacts will also depend on partisan identity. Using a large sample of survey data, Mosier and Rimal (2020) find that Democrats are significantly more likely to report a vegan or vegetarian-based diet compared to Republicans. This could be because conservatives in the U.S. are more attached to meat compared to liberals due to feeling less concern about social justice and less supported socially for diet changes (Hodson and Earle, 2018). Another possible explanation concerns “food neophobia” or the reluctance to eat unfamiliar foods, which is higher among conservatives (than liberals) because they tend to hold more negative attitudes toward those outside their social identity (Guidetti et al., 2022). This partisan underpinning of identification with meat is playing out in U.S. national politics with more fervor than ever before: “I will NEVER eat one of those FAKE burgers made in a LAB. Eat too many and you'll turn into a SOCIALIST DEMOCRAT. Real BEEF for me!!” (Tweet on November 5th 2022 from Representative Ronny Jackson, Texas's 13th district).

Political attention on (farm) animal rights may also engender general voter backlash, with stronger effects along racial lines and on the right than the left in the United States. The anticipation of backlash, particularly from minoritized groups, is a key driver of why there is virtually no discussion of animal rights in American national politics. The reason for this may be connected to negative perceptions of the animal protection movement as racially homogeneous or insensitive (Kymlicka and Donaldson, 2014; Wrenn, 2016), comprised by mostly white and middle-class participants (Maurer, 2010), and failing to adequately address racial inequity within it (Harper, 2010; Reisman et al., 2021). Despite the Left's disavowal of animal rights, the vast majority of vegans who choose to eschew animal-based foods for ethical reasons are left-leaning (Wrenn, 2017). More generally, recent empirical work reveals differences across Americans on favorability toward animal rights issues, with Democrats and Democratic-leaning Independents being much more supportive overall than Republicans and Republican-leaning Independents (Riffkin, 2015; Park and Valentino, 2019). This means that voter backlash against a political candidate who puts attention on animal rights, while generally the case independent of partisanship, may be harsher for Republicans in particular.

### Insights from psychology: other kinds of voter evaluations

A “nascent, fast-growing body of work” in psychology finds that social identification does not stop at the species border (Dhont et al., 2019, p. 773). The extent to which humans identify with the human in-group and are hostile toward the non-human (animal) out-group varies across individuals (Amiot and Bastian, 2017; Auger and Amiot, 2019; Caviola et al., 2019). Similar to other group-based social dynamics, backlash occurs when an individual deviates from human in-group norms, like those of anthropocentrism, a set of attitudes, beliefs, and standards that defines an arbitrary and implicit inter-species hierarchy on earth, which strongly favors the interests of the most dominant and powerful species (human) (Saha, as quoted in Hindin, 2022).

One line of research in this body of work has explored social backlash faced by vegans who deviate from anthropocentric norms because they are less hostile toward the non-human out-group (Minson and Monin, 2012; Earle and Hodson, 2017; MacInnis and Hodson, 2017; Judge and Wilson, 2019; Stanley, 2021). Personal motivations matter; vegans who cite animal rights or environmental concern as a basis for renouncing meat are viewed as particularly threatening compared to vegans who are motivated by health reasons (MacInnis and Hodson, 2017; Hodson et al., 2019). This is because individuals who deviate from majority group norms for ethical reasons pose a challenge to the group's positive evaluation of itself (Cramwinckel et al., 2015)—for e.g., individuals derive a positive self-concept from being a part of a species or group that is seen as “good” as opposed to “cruel” or “environmentally destructive”.

This body of work highlights two main norms associated with human group membership: (1) only humans matter morally and that (2) human dominance over nature and other animals is absolute (see also White, 1967; Naess, 1986). Violations of these standards by vegans and vegetarians who eschew some or all animal products can lead to negative judgments about their morality and strength, in particular (Minson and Monin, 2012; Judge and Wilson, 2019; see also Greenebaum and Dexter, 2018 for connection between high meat consumption and masculine norms of power/dominance). However, to the author's knowledge, these theories have not been tested in a political or election context. For this reason, I also measure other voter evaluations of the hypothetical political candidate in Study 1 as “immoral” or “weak” in order to understand what particular perceptions could be driving voter backlash. To the extent that a political candidate who puts political attention on either farm animal rights or meat's environmental costs can trigger similar evaluations as vegans and vegetarians do, then I expect that voters perceive such a candidate as morally deviant or less dominant.

Another line of research in this body of work explores meat-related cognitive dissonance that can trigger “dislike” of targets who remind individuals that their values (for e.g., care for animals) and actions (for e.g., meat eating) do not align. When a dissonant state occurs, affected individuals can either change their behavior or use dissonance reduction strategies like disassociation of meat from its animal origins (Kunst and Hohle, 2016) and denial/rationalization (Bastian et al., 2012; Piazza et al., 2015). Most relevant to candidate evaluation is an indirect perceptual strategy of dissonance reduction called *do-gooder derogation*, which leads to backlash against the individual who triggers the dissonant state (Minson and Monin, 2012). Since this “kill the messenger” effect can occur simply by raising the topic of meat reduction or farm animals (Rothgerber, 2020), I expect that voters perceive a political candidate who puts political attention on either farm animal rights or meat's environmental costs as less likable. To the author's knowledge, a test of do-gooder derogation (resulting from meat-related cognitive dissonance) in a political or election context has not been conducted.

Study 2, which uses a conjoint experiment that facilitates intersectional analysis relevant to this topic, builds on this human-animal intergroup dynamics literature directly by exploring how diverse political leaders with characteristics like a vegan dietary preference, strong support for animal rights, and personal concern for farm animals in particular fare in national elections. The experimental work discussed in this section has only considered backlash at the individual level, so a key theoretical contribution of this paper is to assess how these attributes, when explicitly presented, impact voter support at the leader level in a political context. Since political candidates with such attributes can violate human in-group norms and may also trigger do-gooder derogation, I expect less voter support for them, all else equal, while noting that there might be variation based on their gender and/or race, and respondent party affiliation.

## Study 1

The vignette experiment investigates how political attention on transportation's environmental costs, meat's environmental costs, and farm animal rights impacts the likelihood of voter support for a hypothetical U.S. presidential primary candidate. In addition, I assess other voter evaluations like the perceived morality, dominance, and likeability of the political candidate to explore what underlying perceptions could be driving potential voter backlash against someone who violates the norms of human group membership in an election context.

### Method

NORC, at the University of Chicago, fielded Study 1 using the AmeriSpeak probability-based panel, designed to be representative of the U.S. household population. A total of 2,116 U.S. citizens were randomly sampled from a national panel, to which is recruited participants using U.S. mail, telephone, and in-person methods. Pursuant to the recommendations of Kane and Barabas (2019), a factual manipulation check was used to test whether the treatment manipulations conducted in the experiment were perceived by the subjects. Such a test is particularly important when the treatment stimuli require that participants read carefully (Kane and Barabas, 2019), as the vignettes used in this experiment do. A total of 244 subjects were dropped from the final dataset for failing the factual manipulation check. The final sample, therefore, included 1,872 U.S. citizens. The vignette experiment was conducted online via NORC's in-house survey platform. Descriptive statistics and other details about question wording for both Study 1 and 2 can be found in Section 1 of the Supplementary material. I note that the main results for Study 1 hold for the full sample of respondents (*N* = 2,116) as well.

#### Design

Subjects were presented with one of four versions of a stump speech from a hypothetical political candidate running in a presidential primary. The experiment had, in total, 4 conditions (i.e., versions of the stump speech): 1 control and 3 experimental. The control version of the stump speech was adapted from a speech written by two former political speechwriters, Republican and Democrat, hired by 538, a poll analysis website, to write, “The Perfect Presidential Stump Speech” (Swaim and Nussbaum, 2016). Each treatment added just one paragraph to the stump speech used in the control condition. Political attention on meat's environment costs was cued with the following paragraph [the “Meat (Environment)” condition]: “On the environment, we know that meat consumption plays a huge role in climate change. And, that's why it's time for us to work together as a nation to reduce our reliance on meat and dairy and focus on solutions like plant-based foods and artificial meats instead.” Political attention on transportation's environmental costs was cued with [the “Transportation (Environment)” condition]: “Finally, on the environment, we know that transportation plays a huge role in climate change. And, that's why it's time for us to work together as a nation to reduce our reliance on gasoline-powered vehicles and focus on solutions like public transportation and electric cars instead.” Finally, political attention on farm animal rights was cued with the following additional paragraph (the “Animal Rights” condition): “Finally, on animal rights, we know that animals deserve proper protection. Our nation should work toward clearly defining the limits of how animals—particularly farm animals—may be treated and what they can be used for.”

For all versions of the speech, respondents were asked to consider a hypothetical presidential primary candidate named “Tom Larson,” thereby holding gender and race constant across the experimental conditions in Study 1. It is most likely that respondents assumed that “Tom Larson” was white and male identifying (Petsko and Rosette, 2022). Following the speech, respondents were asked how likely they would be to vote for the candidate to be a nominee for President within their party using a 7-point Likert scale. A note was added for those who do not identify with a political party: “If you do not identify as a Republican or Democrat, please evaluate the primary candidate as a potential presidential nominee who you might consider.” Respondents were also asked to rate the likeability, morality, and dominance (“weakness”) of the political candidate using an 11-point Likert scale. Finally, respondents answered a factual manipulation check question, which served as both an attention test and confirmation that the treatment had the intended effect (Kane and Barabas, 2019).

### Results of Study 1

All analyses were conducted using R 4.2.0 GUI for Mac.

#### Treatment effects on likelihood of voting: diffuse effects

The distributional analysis conducted by political scientists suggests that there is overall broad voter support for governments that put political attention on climate change. Table 1 shows average treatment effects (ATEs) across the experimental conditions in Study 1 (derived from a linear model using survey weights provided by NORC). An ATE can be interpreted as the average causal effect of a particular treatment on the likelihood of voting for the hypothetical political candidate (1–7 scale) compared to the control condition. For political attention on transportation's environmental costs, there were no significant effects on the likelihood of voting for the candidate (compared to the control condition) in the full sample [ATE = 0.088, SE = 0.109, *p* = 0.422, Cohen's d = −0.03]. However, a hypothetical political candidate who puts political attention on meat's environmental costs did face broad voter backlash relative to the control candidate in the full sample of U.S. citizens, though the effect size is small [ATE = −0.579, SE = 0.109, *p* < 0.01, Cohen's *d* = 0.36]. Similarly, even when compared to the transportation condition, political attention on meat led to significant voter backlash [ATE = −0.666, SE = 0.112, *p* < 0.01, Cohen's *d* = 0.39; Supplementary Table 3].

**Table 1:** Average treatment effects (all respondents).

	**Dependent variable**
	**Support**
Meat (environment)	−0.579^***^
	(0.109)
Animal rights	0.060
	(0.108)
Transportation (environment)	0.088
	(0.109)
Observations	1,870
R^2^	0.025
Adjusted R^2^	0.023

The results are relative to the control condition. ^*^p < 0.1; ^**^p < 0.05; ^***^p < 0.01.

The work in political theory on animal rights indicates that, overall, political attention on animal rights could bring about general voter backlash. Surprisingly, in Study 1, a hypothetical political candidate who puts political attention on farm animal rights faced no electoral backlash compared to the candidate in the control condition [ATE = 0.060, SE = 0.108, *p* = 0.578, Cohen's d = −0.02]. This finding also held for the animal rights condition when compared to the transportation condition [ATE = −0.028, SE = 0.112, *p* = 0.805, Cohen's d = −0.01].

#### Conditional treatment effects on likelihood of voting: concentrated effects

Unfortunately, the NORC dataset did not provide information to accurately determine *where* fossil fuel phaseout costs would be concentrated for the entire country. For the phaseout costs associated with meat reduction, it is mainly those in rural areas who would be most negatively impacted. Table 2 presents conditional average treatment effects (CATEs) by respondent region for the meat (environment) condition. A CATE, in this case, is the average causal effect of the meat (environment) treatment on the likelihood of voting for the hypothetical political candidate compared to the control, conditioned on whether the respondent lives in an urban or rural area (as defined by the US Census Bureau). For urban respondents, political attention on meat's environmental costs led to a 0.43 point (average) drop in voter support [SE = 0.123, *p* < 0.01, Cohen's *d* = 0.32]. For rural respondents, this kind of political attention led to a much larger average drop in voter support [CATE = −1.354, SE = 0.283, *p* < 0.01, Cohen's *d* = 0.58]. The difference between these respondent subgroups holding the experimental condition (meat) is also significant (*p* < 0.01; Supplementary Table 4).

**Table 2:** Conditional average treatment effects (urban/rural).

	**Dependent variable**	
	**Support**	
	**(Urban respondents)**	**(Rural respondents)**
Meat (environment)	−0.427^***^	−1.354^***^
	(0.123)	(0.283)
Observations	789	138
R^2^	0.015	0.144
Adjusted R^2^	0.014	0.138

The results are relative to the control condition. ^*^p < 0.1; ^**^p < 0.05; ^***^p < 0.01.

The lack of political attention on animal rights might be explained, in part, by identity politics: a concern that including animal rights on the agenda might compete with or trivialize the interests of disadvantaged human subgroups. Table 3 shows CATEs for Black and White respondents in the animal rights condition. There was no significant effect of the animal rights treatment on voter support compared to the control for either subgroup. I also checked for effects in this experimental condition for Non-White Hispanic respondents in the sample. There was no significant effect of the animal rights treatment on voter support for this group of respondents either (Supplementary Table 5). Finally, I considered any differences among these three racial/ethnic groups in the sample for the animal rights condition and found no significant interactive effects for any (Supplementary Table 6).

**Table 3:** Conditional average treatment effects (Black/White respondents).

	**Dependent variable**	
	**Support**	
	**(Black respondents)**	**(White respondents)**
Animal rights	−0.316	0.166
	(0.352)	(0.131)
Observations	77	649
R^2^	0.011	0.002
Adjusted R^2^	−0.003	0.001

The results are relative to the control condition. ^*^p < 0.1; ^**^p < 0.05; ^***^p < 0.01.

#### Conditional treatment effects on likelihood of voting: party effects

Political psychologists highlight the polarizing role of party identification when it comes to environmental politics. Table 4 presents CATEs for Republican and Democratic respondents across the experimental conditions. For Democrats, political attention on meat's environmental costs had no significant impact on voter support (compared to the control). For Republicans, however, political attention on meat's environmental costs led to a large average drop of 1.08 points on the 1–7 voter support scale [SE = 0.192, *p* < 0.01, Cohen's *d* = 0.67]. Holding the meat (environment) condition, the difference between Republicans and Democrats is significant (*p* < 0.01; Supplementary Table 7). Turning to the animal rights condition, there were no significant effects of this treatment on voter support for either Republicans or Democrats in the sample. Finally, political attention on transportation's environmental costs led to a vote bump of +0.44 points on average for Democrats [SE = 0.150, *p* < 0.01, Cohen's d = 0.25] but backlash from Republicans in the sample [CATE = −0.331, SE = 0.169, *p* < 0.1, Cohen's *d* = 0.22]. Holding the transportation (environment) condition, the difference between Republicans and Democrats is significant (*p* < 0.01; Supplementary Table 7).

**Table 4:** Conditional average treatment effects (Democrats/Republicans).

	**Dependent variable**	
	**Support**	
	**(Democratic respondents)**	**(Republican respondents)**
Meat (environment)	−0.203	−1.079^***^
	(0.157)	(0.192)
Animal rights	0.226	−0.110
	(0.158)	(0.171)
Transportation (environment)	0.443^***^	−0.331^*^
	(0.150)	(0.169)
Observations	478, 456, 471 (in order from top)	341, 377, 368
R^2^	0.003, 0.004, 0.018	0.085, 0.001, 0.010
Adjusted R^2^	0.001, 0.002, 0.016	0.083, −0.002, 0.008

The results are relative to the control condition. ^*^p < 0.1; ^**^p < 0.05; ^***^p < 0.01.

#### Average treatment effects on other voter evaluations: exploring perceived morality, dominance, and likeability

Insights from psychology point to key social and cognitive mechanisms that could underpin voter backlash against a candidate who puts political attention on animal issues. Table 5 shows the ATEs of the animal-related experimental conditions (compared to the control) across three models with different dependent variables (0–10 scale for all): perceived morality, dominance, and likeability of the hypothetical candidate in Study 1. Political attention on meat's environmental costs reduced the likeability of the political candidate [ATE = −0.794, SE = 0.147, *p* < 0.01, Cohen's *d* = 0.38], but there were no significant effects on perceived morality or dominance. On the other hand, political attention on farm animal rights reduced the perceived morality of the candidate [ATE = −0.472, SE = 0.170, *p* < 0.01, Cohen's *d* = 0.12], but there were no significant effects on perceived dominance or likeability. Given the strong effects of respondent party identification in the earlier section, I checked for heterogeneous effects for each of these findings: effects on both perceived morality and likeability of the candidate in the animal rights and meat (environment) conditions, respectively, were more negative for Republicans than Democrats (*p* < 0.05; see Supplementary Table 8).

**Table 5:** Average treatment effects (all respondents, other voter evaluations).

	**Dependent variable**		
	**Morality**	**Dominance**	**Likeability**
	**(1)**	**(2)**	**(3)**
Meat (environment)	−0.011	0.244	−0.794^***^
	(0.171)	(0.176)	(0.147)
Animal rights	−0.472^***^	−0.171	−0.088
	(0.170)	(0.174)	(0.146)
Observations	1,372	1,376	1,387
R^2^	0.007	0.004	0.024
Adjusted R^2^	0.006	0.002	0.023

The results are relative to the control condition. ^*^p < 0.1; ^**^p < 0.05; ^***^p < 0.01.

### Discussion

The results of Study 1 challenge the conventional wisdom among some political scientists that climate policy in the United States enjoys broad public support by experimentally testing the effects of political attention on addressing environmental costs in two comparable sectors: meat and transportation. Previous studies that have shown that American voters are largely supportive of climate policy have either relied on small sample observational data (Carmack et al., 2022 compared voter results between two election cycles, with the second post-COVID) or public opinion survey data (Aldy et al., 2012; Ansolabehere and Konisky, 2016). While it may be the case that Americans are supportive, in theory, of some climate policies, it is in elections when most take action through voting and evaluating candidates based on their political agendas. This experimental work shows that overall voter support depends on the particular environmental issue area in question. Climate policy focused on the meat sector in the U.S. likely would not have broad or diffuse public support; instead, there may be voter backlash, with even stronger negative effects where the phase-out costs would be concentrated (in rural agricultural areas). For transportation and climate policy, the results show that, overall, voters are neutral in elections.

Partisan identification, however, did seem to play a strong role in predicting voter support for political candidates who center the environment. The results of Study 1 demonstrated strong differences by respondent party, with Democrats significantly more supportive of political attention on the environmentally costly meat and transportation sectors than Republicans. These findings are in line with research indicating that political orientation is a decisive factor in Anglo-Saxon countries where left-wing governments are significantly more likely to pass climate friendly laws relative to right-wing governments (Fankhauser et al., 2015). Study 1 contributes to this literature by showing that there is variation in levels of support (and backlash), dependent on the environmental issue area. Most notably, Democrats provided a boost to a political candidate who brings up transportation's environmental costs, but they did not provide a similar boost to a political candidate who puts meat's environmental costs on the agenda. Left-leaning voters may exhibit inconsistency in their support for climate policy depending on the target sector. This implies that the salience of the food and climate change nexus is low among Democratic voters, which is corroborated by a recent representative survey that shows that most people across 5 country contexts (including the U.S.) don't see industrial meat as a key cause of global warming (Madre Brava, 2023), something that can be addressed through greater media coverage, education campaigns, and better scientific communication.

The surprising lack of voter backlash against a political candidate who centers farm animal rights on their agenda on the left and right is in line with the results of recent state-level referendums in the United States. The lack of federal animal welfare legislation has moved the focus of animal welfare groups to the state level, where 12 states have passed laws to protect farm animals via referendums, often with large majorities and strong bipartisan support (Vogeler, 2020). While the results of Study 1 showed no differences in voter support across respondent racial/ethnic groups, the sample sizes were small for Black (*N* = 77) and Non-White Hispanic subjects (*N* = 130) in the animal rights experimental condition.

Finally, political attention on animals affected other voter evaluations, but how depended on the framing. Backlash due to violations of anthropocentric norms (i.e., the candidate as “morally deviant”) occurred only against the hypothetical political candidate who puts political attention on farm animal rights, and it was driven by Republicans; nevertheless, it did not seem to translate to *voter backlash*. Only in the meat (environment) condition was the political candidate rated as less likable (compared to the control; again, driven by Republicans), an outcome associated with do-gooder derogation. However, Study 1 did not directly test that meat-related cognitive dissonance had, in fact, been triggered by the stimuli. In addition, it is unclear how these other voter evaluations impact voter support because the research design did not enable mediation analysis and so all outcomes were modeled separately.

## Study 2

The conjoint experiment further explores the surprising results of Study 1 (i.e., no backlash from Republicans or Democrats for political attention on farm animal ethics) by directly testing candidate characteristics like veganism, concern for farm animals, and personal support for animal rights to measure their effects on (forced) vote choice. Unlike vignette experiments, conjoints enable independent randomization of multiple variables within a single experiment (Hainmueller et al., 2013). As a result, Study 2 adds valuable and relevant insight into how less anthropocentric candidates with different gender and racial identities might fare in national elections. Given the significance of identity group politics to the exclusion of animal rights on the political agenda, intersectional analysis is key to understanding voter evaluations of those political actors who are most likely to put attention on farm animals.

### Method

Dynata fielded the conjoint experiment used in Study 2 to their panel, which is the largest first-party one in the world. They recruited a total of 857 U.S. citizens. This sample is not probability-based, but it was balanced to be representative of the U.S. population on age, gender, ethnicity, region (all based on Census data), and partisan affiliation (based on a recent Gallup poll).

**Figure 1:**
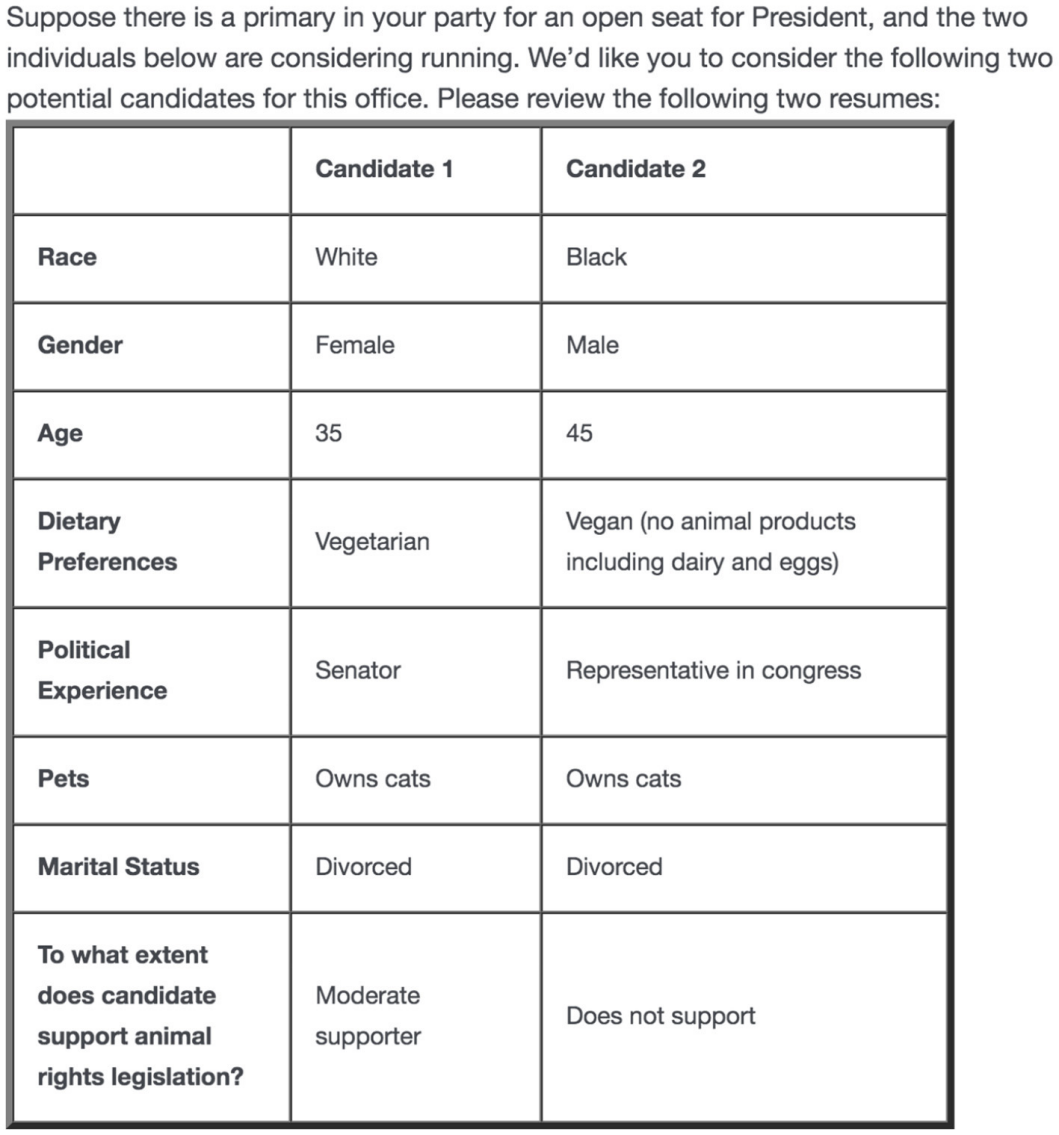
Design for the conjoint experiment (what a respondent might have seen).

Respondents in Study 2 were presented with five hypothetical presidential primary elections and asked to consider the candidates within their party. Each election involved a table with two candidate resumes including information about each candidate's dietary preference (none, vegetarian, or vegan), pets (no pets, cats, dogs, or rescued farm animals), personal support for animal rights (does not support, moderate supporter, or strong supporter), race (White, Latino/a, or Black), and gender (man or woman). Other relevant attributes were also included in the table to create a full candidate profile, like age (35, 45, 55, or 65), marital status (single, married, or divorced), and previous political experience (no experience, Mayor, Representative in Congress, or Senator). Attributes for each candidate were independently and randomly selected from the set of options for each characteristic. Figure 1 presents an example of what a respondent might have seen. After each table, respondents were asked to pick which candidate of the two they would be most likely to vote for in the presidential primary.

Though researchers have found that respondent selections in conjoints do mirror real-life preferences (Hainmueller et al., 2015), this study reduces the artificiality of the experiment even further through a small deception, implemented before the survey starts, which led participants to believe that the candidates in the tables they were about to view were real people working with a political recruitment organization. This methods innovation promotes greater cognitive engagement with the task by enhancing the overall authenticity of the tables and scenario.

### Results of Study 2

All analyses were conducted using R 4.2.0 GUI for Mac.

#### Determinants of vote choice

**Figure 2:**
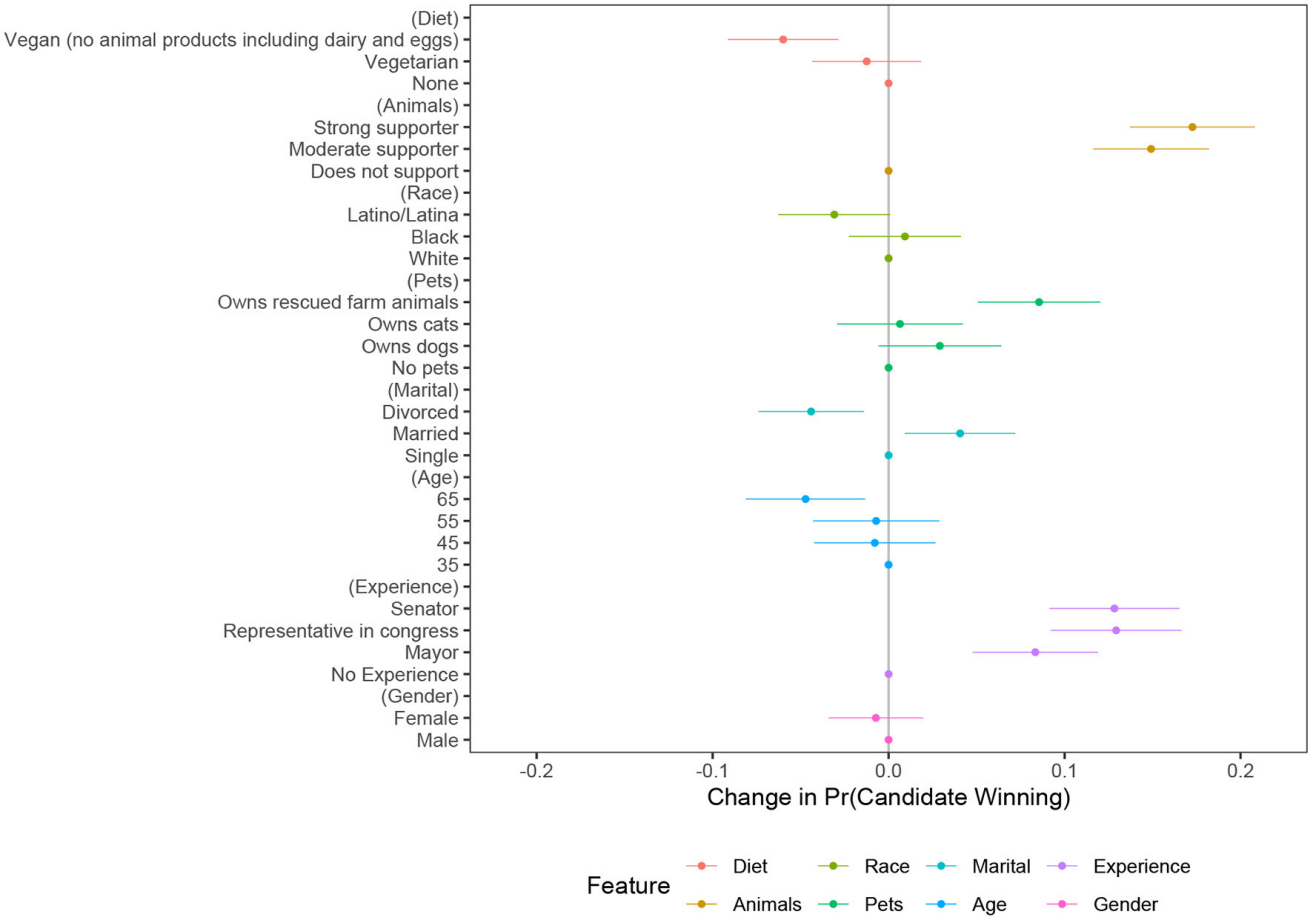
Determinants of vote choice. Figure shows percentage point change in probability of winning. Sample size *N* = 857 respondents, or at candidate level *N* = 8,570. 95% confidence intervals are represented by bands around plotted estimates.

Figure 2 presents results for the determinants of voice choice for the full sample of respondents in Study 2. The quantity of interest is the Average Marginal Component-specific Effect (AMCE), which is the treatment effect of a particular attribute level (compared to the base level) averaged over the joint distribution of all other attribute values (Hainmueller et al., 2013). I cluster standard errors at the respondent level to account for within-election variation. I find that, contrary to expectations and in line with the results of Study 1, a candidate who has rescued farm animals or strongly supports animal rights was more likely to win their election, all else equal. Compared to a candidate who does not support animal rights, a strong supporter enjoyed a large 17.3% boost (*p* < 0.01) in the probability of winning. Similarly, compared to a candidate who has no pets, one who owns rescued farm animals got a bump of almost 9% points (*p* < 0.01) in the likelihood of winning their election. In contrast, a vegan candidate was less likely to win compared to a candidate who has no dietary restrictions (−6%, *p* < 0.01).

#### Determinants of vote choice by respondent party affiliation (Democrats and Republicans)

**Figure 3:**
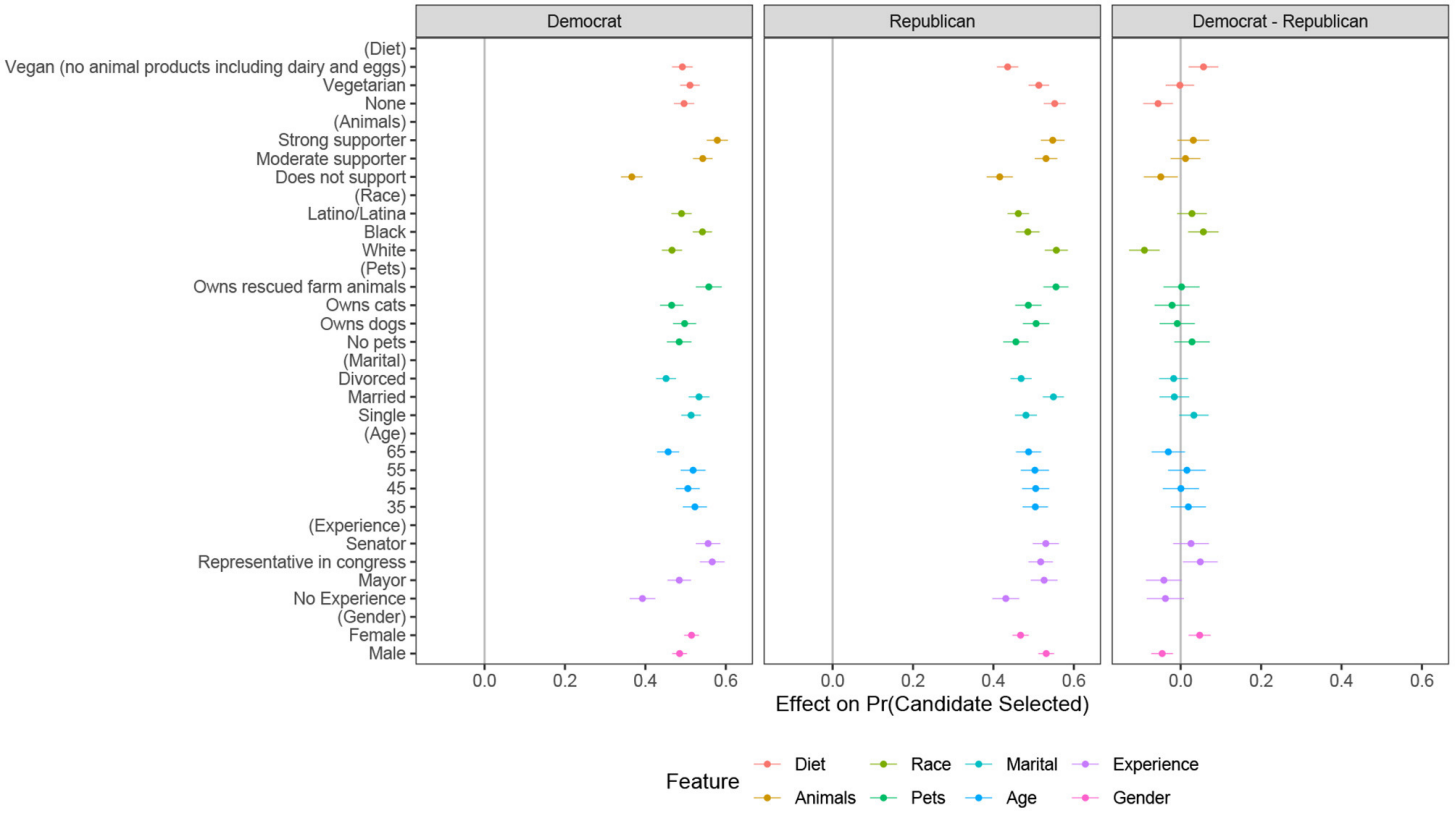
Conditional marginal means by respondent party ID. Figure shows favorability toward candidate profiles for respondents identifying as Democrat, Republican, and the difference using conditional marginal means. Sample size *N* = 3,970 Republicans and 4,540 Democrats, or at candidate level *N* = 8,510. 95% confidence intervals are represented by bands around plotted estimates.

Comparisons of AMCEs between subgroups of respondents can lead to unclear inferences because these estimates depend on the arbitrary selection of attribute baselines by the researcher (Leeper et al., 2020). For this reason, Figure 3 plots the conditional marginal means (MMs) by respondent party affiliation and the differences in these means (far right panel). MMs can be interpreted as the probability of a respondent selecting a candidate with a particular attribute level. There were no significant differences between Democrats and Republicans regarding preferences for candidates who have rescued farm animals or who strongly support animal rights. On the other hand, there was significant voter backlash against vegan candidates driven by Republicans, who were less likely to vote for such candidates compared to Democrats (MMs for Democrats and Republicans, respectively, were 0.49 and 0.43; a difference of 6% points, *p* < 0.01).

#### Most preferred candidate profiles for Democratic and Republican respondents

The hypothetical candidate in Study 1 was always “Tom Larson” due to the difficulty of implementing multiple treatments using a vignette design. Thus, a key purpose of using a conjoint experiment in Study 2 is to conduct intersectional analysis and present results by candidate race and gender. Tables 6, 7 show the top 10 profiles of candidates most likely to win their elections for Democrats and Republicans in the sample, respectively. To identify these top profiles, I estimated Average Marginal Interaction Effects (AMIEs). AMIEs are non-parametrically estimated using ANOVA regression with weighted zero-sum constraints, enabling the estimation of predicted treatment effects for a large number of joint attribute values or candidate profiles (Egami and Imai, 2018). Democrats most favored Latina and Black women candidates who are strong animal rights supporters (top 8 of 10 ranked profiles) and have rescued farm animals (overall 8 of 10 ranked profiles). Republicans most favored White men candidates who support animal rights (strongly or moderately, for all 10 ranked profiles) and have rescued farm animals (overall 4 of 10 ranked profiles and most frequently ranked, including in the top 2 most preferred profiles).

**Table 6:** Profiles of candidates who are most likely to win among democrats.

**Rank**	**Treatment effect**	**Race and gender of candidate**	**Diet**	**Animal rights**	**Pets**
1	0.144270138	Black woman candidate	Vegetarian	Strong supporter	Owns rescued farm animals
2	0.144270138	Black woman candidate	None	Strong supporter	Owns rescued farm animals
3	0.142227958	Black woman candidate	Vegan	Strong supporter	Owns rescued farm animals
4	0.120972308	Latina candidate	None	Strong supporter	Owns rescued farm animals
5	0.120972308	Latina candidate	Vegetarian	Strong supporter	Owns rescued farm animals
6	0.118930127	Latina candidate	Vegan	Strong supporter	Owns rescued farm animals
7	0.105088686	Black woman candidate	Vegetarian	Strong supporter	Owns dogs
8	0.105088686	Black woman candidate	None	Strong supporter	Owns dogs
9	0.103766048	Black woman candidate	None	Moderate supporter	Owns rescued farm animals
10	0.103766048	Black woman candidate	Vegetarian	Moderate supporter	Owns rescued farm animals

Results come from analysis of predicted values for unique treatment combinations using the FindIt package for R version 4.2.0 (Egami et al., 2018).

**Table 7:** Profiles of candidates who are most likely to win among republicans.

**Rank**	**Treatment effect**	**Race and gender of candidate**	**Diet**	**Animal rights**	**Pets**
1	0.11615711	White man candidate	None	Strong supporter	Owns rescued farm animals
2	0.11615711	White man candidate	None	Moderate supporter	Owns rescued farm animals
3	0.09345981	White man candidate	None	Moderate supporter	Owns dogs
4	0.09345981	White man candidate	None	Strong supporter	Owns dogs
5	0.09205777	White man candidate	Vegetarian	Strong supporter	Owns rescued farm animals
6	0.09205777	White man candidate	Vegetarian	Moderate supporter	Owns rescued farm animals
7	0.07969924	White man candidate	None	Strong supporter	Owns cats
8	0.07969924	White man candidate	None	Moderate supporter	Owns cats
9	0.0555999	White man candidate	Vegetarian	Strong supporter	Owns dogs
10	0.0555999	White man candidate	Vegetarian	Moderate supporter	Owns cats

Results come from analysis of predicted values for unique treatment combinations using the FindIt package for R version 4.2.0 (Egami et al., 2018).

The seemingly very strong preference of Democrats in the sample for women of color candidates who demonstrate personal concern for farm animals and support animal rights more generally warrants further investigation. Considering only those hypothetical candidates who either strongly support animal rights or have rescued farm animals, Supplementary Table 9 presents these AMCEs by candidate gender and race for all Democratic respondents. Compared to a White woman candidate who strongly supports animal rights, Democrats gave a boost of 14.9% points (*p* < 0.01) to a Black woman candidate who similarly strongly supports animal rights. For Latina candidates, this boost was 17.2% points (*p* < 0.01). In terms of pet ownership, Democrats preferred a Black woman candidate who owns rescued farm animals compared to a White woman candidate who also owns rescued farm animals by +14.1% points (*p* < 0.05). For Latina candidates who own rescued farm animals, Democrats were 13.7% points (*p* < 0.05) more likely to vote for her (compared to a similar White woman candidate).

### Discussion

Study 2 is consistent with the surprising result of Study 1: a national-level political candidate who is personally concerned for farm animals or strongly supports animal rights (more generally) received a significant bump in voter support. This is true independent of respondent party identification, implying there is taste on both the left and the right in the United States for animal-friendly political candidates. A possible explanation is that these attributes cue higher levels of perceived empathy, humanizing political leaders through demonstrative concern for the powerless (Pycior, 2005; Everett et al., 2019). But, such a vote bump did not extend to a vegan political candidate who did face backlash from Republican respondents (Democrats were neutral). The finding that conservatives are more likely to socially punish vegans (Dhont and Hodson, 2014; Judge and Wilson, 2019) may likely extend to the leader level on the right.

The results of Study 2 also show that the race and gender of animal-friendly candidates mattered for Republicans and Democrats. Republicans most preferred white men candidates who are animal-friendly, while Democrats provided the biggest vote bumps to women of color candidates with such attributes. Hayes (2005) argues that Republican candidates are perceived to be stronger, more moral leaders, while Democrats have the advantage in perceived compassion and empathy due to each party's ownership of different issue areas (e.g., defense for Republicans and social welfare for Democrats). Overall, voters perceive Black politicians as more empathetic (Gordon and Miller, 2005), even though Republican voters tend to generally prefer White candidates compared to Democratic voters (Crowder-Meyer et al., 2021). It is possible that trait-issue ownership by parties and racial cues interact in American politics such that the electoral benefits of higher perceived candidate empathy (owned by Democrats) is greatest for women of color candidates on the left and white men candidates on the right. Another possibility is that Democratic voters are more sensitive to the concern that the inclusion of animal rights competes with or trivializes the interests of minoritized groups like African-Americans or Non-White Hispanics, so they tend to feel more comfortable supporting animal-friendly candidates from these racial/ethnic groups. Unfortunately, Study 2 is limited in the extent to which it can provide a clear explanation of the underlying mechanisms since it only measures one outcome (vote choice). Furthermore, the sample sizes for Black and Non-White Hispanic subjects were too small in Study 2 to investigate differences in voter preferences by respondent racial/ethnic group (like in Study 1).

## General discussion

Study 1 revealed that national-level Democratic presidential primary candidates in the United States may not have to worry about voter backlash if they put attention on the country's need to reduce its reliance on environmentally costly animal-based foods. A logical next step for this line of research on how voters react to meat on the political agenda is to extend the findings using a general election setting and other country contexts outside the United States. In the United States, partisan rancor has already emerged around meat (for a good summary, see Smith, 2021), despite meat's glaring omission from key pieces of environmental legislation like the 2022 Inflation Reduction Act. Future research in political psychology on meat politics can hone in on testing framing strategies that inoculate political actors during general elections against extremist right-wing claims, like the one that “[Democrats] are trying to take hamburgers away” (Sebastian Gorka, Conservative Political Action Conference on February 28, 2019).

Outside of the U.S. context, there has been variation in how public audiences have reacted to proposed national policies to reduce meat consumption. In Germany, meat consumption has decreased significantly since 2020 due to, in part, political attention on the need to shift diets from key political leaders like Cem Özdemir, the Minister of Food and Agriculture (Torrella, 2022). But, in the Netherlands, government plans to reduce the number of livestock in the country by a third were met with strong opposition from farmers, leading to an unexpected sweeping win by a small pro-farmers party in the 2023 local elections that upset the liberal (pro-environment) ruling party (Coates, 2023). Future research might explore further the important role of coalition building, which was largely absent in the Netherlands case, as well as the electoral trade-offs associated with more stringent, heavy-handed policies vs. softer approaches (like in Germany where political attention tied to changes in the food environment seems to have already made a difference).

The successful inclusion of animal rights and animal-friendly politicians to national-level politics could yield environmental and ethical benefits by shaping social norms around the need to reduce meat consumption from the top-down. While there may be social backlash against individuals who violate anthropocentric norms, based on Study 1 and 2, it does not appear that this backlash extends to the leader level for (farm) animal rights in a voting context. This could be due to a leader's unique ability to escape social costs associated with deviation from in-group norms, which can, in some cases, lead to social innovation and progress (Moscovici and Lage, 1976; Abrams et al., 2018). For example, in Study 1, even though respondents did find the political candidate who put attention on farm animal rights to be morally deviant—evidence of deviation from group norms—they were not less likely to vote for him. These findings align with recent research (published in *Nature*) showing that, in Germany, animal welfare concerns are a stronger determinant of public support for meat taxation than climate change mitigation (Perino and Schwickert, 2023). A next step in research is to identify the precise causal mechanisms at the leader level that underpin these findings, potentially using a parallel mediation design (Imai and Yamamoto, 2013) to explore perceived empathy, in particular. Multiple studies find a link between moral concern for animals and higher levels of internal empathy (Kessler et al., 2016; Caviola et al., 2018; Rosenfeld, 2018). But, whether this translates to higher perceived empathy, a powerful factor in elections on both sides of the aisle (Laustsen and Bor, 2017; McDonald et al., 2019), is unknown.,

## Summary and conclusion

In this study, I evaluated how voters reacted to political attention on animals and animal-friendly candidates of diverse backgrounds running in hypothetical U.S. presidential primaries using two different kinds of experimental methods. I found that, overall, political attention on the need for climate policy that addresses meat's environmental costs triggered a small level of voter backlash. At the same time, voter reactions were strongly determined by partisan affiliation: Democrats were neutral, but there was strong voter backlash from Republicans. On the other hand, I found no evidence of voter backlash, either on the left or right, for political attention on farm animal rights. A second study added to this finding by testing how diverse candidates who are animal-friendly fared in elections. While there was overall voter support for candidates who are personally concerned for farm animals and strong animal rights supporters (without significant differences by party), the gender and race of candidates mattered differently for Republicans and Democrats.

## Data availability statement

The datasets presented in this study can be found in online repositories. The names of the repository/repositories and accession number(s) can be found below: https://doi.org/10.7910/DVN/ZDT7TW.

## Ethics statement

The studies involving human participants were reviewed and approved by Harvard's Committee on the Use of Human Subjects. The patients/participants provided their written informed consent to participate in this study.

## Author contributions

SS conducted all the research and wrote this paper.

## Footnotes

^1^ https://www.fao.org/gleam/en/

^2^Based on data from the U.S. Department of Agriculture's Economic Research Service, “Major Uses of Land in the United States.” https://www.ers.usda.gov/data-products/major-land-uses/.

^3^ https://gfi.org/wp-content/uploads/2021/09/GFI-Strategic-Plan-v.7-Aug-2021-1.pdf

^4^Based on conversations with or questions posed to Good Food Institute on January 18th 2022, Animal Justice on April 27th 2022, Social Compassion in Legislation on May 5th 2022, a political candidate running for office at the state level (New England) on April 11th 2022, and The Humane League on May 19th 2022.
