# Supplementary material for: Why don't politicians talk about meat? The political psychology of human-animal relations in elections
Source: Front Psychol. 2023 Jun 23;14:1021013. doi: 10.3389/fpsyg.2023.1021013 (PMC10327565; doi:10.3389/fpsyg.2023.1021013)
Supplement: Supplementary file 1 [file Data_Sheet_1.PDF]

Supplementary Materials

Why Don't Politicians Talk About Meat?

Sparsha Saha[1]

Contents

1 Survey Descriptive Statistics, Wording, and Balance **1**
1.1 Wording of Surveys 1
1.1.1 Conjoint Experiment 1
1.1.2 Vignette Experiment 2
1.2 Balance 4

2 Study 1 **6**

3 Study 2 **10**

[1]saha@fas.harvard.edu

1 Survey Descriptive Statistics, Wording, and Balance

Table S1: Summary Statistics for Samples (% or mean/median, where appropriate)

	NORC	SSI	ANES (2016)
Female	52.2%	47.8%	52.0%
Age	49.9	45.5	47.3
Salary	$55,000	$62,500	$63,199
Liberal	29.6%	44%	24%
Conservative	31.1%	55%	36%
White	67.4%	62.4%	74.5%
Black	10.4%	16.4%	12.2%
Hispanic	13.7%	12.7%	10.9%

1.1 Wording of Surveys

1.1.1 Conjoint Experiment

Wording of survey

The survey involved a slight deception, presented at the very beginning of the survey in the consent form. Below is the language:

> "You are being asked to participate in a research study being done by Sparsha Saha from Harvard University. She is working with a DC based political recruitment agency and so the resumes you will see represent actual people taken from a large pool of possible recruits being considered."

This was done to make the context of the survey seem more realistic, since candidate tables in conjoint experiments have been criticized for seeming inauthentic and not reflecting accurately how voters tend to receive information in the real world. Nevertheless, research does suggest that conjoint experiments mirror real-life preferences (Hainmueller, Hangartner and Yamamoto 2015).

Following the consent page, respondents see the following text:

> "For the next few minutes, we are going to ask you to act as if you were about to cast a vote for a candidate running for President within your party.
>
> We will describe to you several pairs of candidates running for this presidential primary. For each pair of people, please indicate which one you would prefer to represent you. Even if you aren't entirely sure, please indicate which of the two you prefer given only the information presented."

Before the first election, respondents see the following text:

> “Suppose there is a primary in your party for an open seat for President, and the two individuals below are considering running. We’d like you to consider the following two potential candidates for this office. Please review the following two resumes: "

After this, the question stem presented was:

> “Based on the limited information above, which of the two candidates would you be more likely to support for President?"

1.1.2 Vignette Experiment

Wording of NORC

The following describes the wording for the vignette experiment (via NORC’s AmeriSpeak Panel).

The general instructions are:

> “You will next be asked to read a short speech by a hypothetical political candidate running for President from your party. If you do not identify with a party, imagine this is a candidate you might consider. After you read this speech, you will then be asked to indicate how you feel about the candidate.”

The instructions right above the text are:

> “Please read the following speech made by a candidate named Tom Larson, who imagine is running in your party’s presidential primary (please read carefully).”

Figure S1 presents the control group speech.

"Thank you all for being here. This is just incredibly humbling. Thank you. For each of us, I believe, there is common ground. And that's the ground we need to cultivate.

On the economy, to fix what's wrong, we have to be honest with ourselves — people are hurting out there. All we need to do is make sure the rules apply to everyone.

On foreign policy, the U.S. military must continue to be the greatest in the world. So, while the Defense Department must justify every line item of taxpayer money, we have to realize that a safe and stable world doesn't come cheap.

On health care, let's fix what's broken and make sure that working Americans can actually access and afford the health care they deserve.

On immigration, we can fix our immigration system and remain a strong, secure nation in the process.

Look, those are just a few of the places where we know we can make progress.

But, for us to do something, I need you to vote. I ask you to stand with me. Join me.

And together, we'll build the country we know we can be. Thank you, and may God bless America."

Figure S1: Control Group Speech

Following the speech, respondents are asked to rate the candidate on three traits (using a 0-10 scale): likeability, morality, and power. The traits are randomized. Figure S2 presents the wording:

Thinking about this candidate, how do the following phrases describe them?

"This candidate is immoral"

"This candidate is likeable"

"This candidate is a weak leader"

Figure S2: Other Voter Evaluations, Question Wording

After this, respondents answer the main outcome question, using a 1-7 scale. Wording below:

> "As above, imagine this candidate is running in a presidential primary election. How likely would you be to vote for this candidate to be a nominee for President? Please use the scale below to indicate."

Finally, respondents are presented a factual manipulation check question. They are asked, "According to the speech you just read, do you remember if the hypothetical political candidate, Tom Larson, discussed any of the following topics? Please check as many as you can remember." The options are: Terrorism, Environment, Diet, Transportation, Animal Rights, and Economy.

1.2 Balance

Table S2: Regression Results

	Dependent variable:						
	Age	Ideology	Gender	Education	Income	Region	Marital Status
	(1)	(2)	(3)	(4)	(5)	(6)	(7)
Treatment 1 (ANIMALS-ENVIRONMENT)	0.121	0.031	0.002	0.006	−0.211	0.120*	0.145
	(0.107)	(0.073)	(0.031)	(0.066)	(0.262)	(0.062)	(0.117)
Treatment 2 (ANIMALS-POLITICS)	0.119	−0.073	0.002	0.065	0.172	0.059	0.044
	(0.106)	(0.072)	(0.031)	(0.065)	(0.259)	(0.061)	(0.116)
CONTROL 2 (TRANSPORTATION)	0.103	0.005	−0.032	0.046	−0.154	0.051	0.083
	(0.106)	(0.072)	(0.031)	(0.065)	(0.259)	(0.061)	(0.116)
Constant	4.902***	2.988***	1.530***	3.279***	10.202***	2.648***	2.528***
	(0.076)	(0.052)	(0.022)	(0.047)	(0.186)	(0.044)	(0.083)
Observations	2,116	2,116	2,116	2,116	2,116	2,116	2,116
R^2	0.001	0.001	0.001	0.001	0.001	0.002	0.001
Adjusted R^2	-0.001	-0.0003	-0.001	-0.001	-0.0001	0.0003	-0.001

Note: *p<0.1; **p<0.05; ***p<0.01

2 Study 1

Table S3: Average Treatment Effects (All Respondents), Compared to Transportation Condition

	Dependent variable:
	Support
Control	−0.088
	(0.109)
Meat (Environment)	−0.666***
	(0.112)
Animal Rights	−0.028
	(0.112)
Observations	1,870
R^2	0.025
Adjusted R^2	0.023
Note:	*p<0.1; **p<0.05; ***p<0.01

Table S4: OLS Regression Estimates (Differences by Respondent Urban-Rural Classification on Voter Support)

	Dependent variable:
	Support
Meat (Environment)	−0.427***
	(0.123)
Rural (Respondent)	0.166
	(0.214)
Meat (Environment):Rural	−0.927***
	(0.307)
Constant	4.323***
	(0.084)
Observations	927
R^2	0.040
Adjusted R^2	0.037
Note:	*p<0.1; **p<0.05; ***p<0.01

Table S5: Conditional Average Treatment Effects (Non-White Hispanics/Whites)

	Dependent variable:	
	Support	
	(Non-White Hispanic Respondents)	(White Respondents)
Animal Rights	0.116	0.166
	(0.297)	(0.131)
Observations	130	649
R^2	0.001	0.002
Adjusted R^2	-0.007	0.001
Note:		*p<0.1; **p<0.05; ***p<0.01

The results are relative to the control condition.

Table S6: OLS Regression Estimates (Differences by Respondent Race/Ethnicity on Voter Support)

	Dependent variable:
	Support
Animal Rights	0.166
	(0.135)
Black (Respondent)	0.985***
	(0.248)
Non-White Hispanic (Respondent)	0.034
	(0.192)
Animal Rights:Black	−0.482
	(0.378)
Animal Rights:Non-White Hispanic	−0.051
	(0.288)
Constant	4.223***
	(0.096)
Observations	935
R^2	0.024
Adjusted R^2	0.012
Note:	*p<0.1; **p<0.05; ***p<0.01

Table S7: OLS Regression Estimates (Differences by Respondent Party on Voter Support)

	Dependent variable:
	Support
Meat (Environment)	−0.203
	(0.152)
Animal Rights	0.226
	(0.156)
Transportation (Environment)	0.443***
	(0.152)
Republican (Respondent)	−0.019
	(0.157)
Meat (Environment):Republican	−0.877***
	(0.233)
Animal Rights:Republican	−0.337
	(0.227)
Transportation (Environment):Republican	−0.774***
	(0.230)
Constant	4.373***
	(0.104)
Observations	1,869
R^2	0.054
Adjusted R^2	0.048
Note:	*p<0.1; **p<0.05; ***p<0.01

Table S8: OLS Regression Estimates (Differences by Respondent Party, Other Voter Evaluations)

	Dependent variable:	
	Morality	Likeability
	(1)	(2)
Meat (Environment)	−0.417*	−0.429**
	(0.240)	(0.209)
Animal Rights	−0.186	0.104
	(0.247)	(0.214)
Republican (Respondent)	0.247	0.142
	(0.248)	(0.215)
Meat (Environment):Republican	0.599	−0.704**
	(0.369)	(0.318)
Animal Rights:Republican	−0.809**	−0.367
	(0.359)	(0.311)
Constant	2.816***	6.509***
	(0.164)	(0.143)
Observations	1,372	1,387
R^2	0.023	0.031
Adjusted R^2	0.018	0.025
Note:	*p<0.1; **p<0.05; ***p<0.01	

3 Study 2

level	estimate	std.error	z	p	lower	upper
White Woman Candidate.Strong supporter	0	NA	NA	NA	NA	NA
White Man Candidate.Strong supporter	0.03175735	0.05453742	0.58230389	0.560362	-0.075134	0.13864872
Black Woman Candidate.Strong supporter	0.14881865	0.05185062	2.87014213	0.00410287	0.0471933	0.250444
Latina Candidate.Strong supporter	0.1720792	0.04953239	3.47407422	0.00051262	0.0749975	0.26916089
Black Man Candidate.Strong supporter	0.13745466	0.04838578	2.84080692	0.00449995	0.04262027	0.23228904
Latino Candidate.Strong supporter	0.06605183	0.05488549	1.20344797	0.22880302	-0.0415218	0.17362541
White Woman Candidate.Owns rescued farm animals	0	NA	NA	NA	NA	NA
White Man Candidate.Owns rescued farm animals	0.08456354	0.06332088	1.33547634	0.1817205	-0.0395431	0.20867019
Black Woman Candidate.Owns rescued farm animals	0.14060798	0.06248528	2.2502576	0.0244326	0.01813908	0.26307688
Latina Candidate.Owns rescued farm animals	0.13706029	0.06442134	2.1275604	0.03337355	0.01079679	0.2633238
Black Man Candidate.Owns rescued farm animals	0.13006518	0.06239205	2.08464362	0.03710166	0.00777902	0.25235134
Latino Candidate.Owns rescued farm animals	0.01143527	0.06105211	0.18730344	0.85142273	-0.1082247	0.13109521

Table S9: Average Marginal Component-specific Effects by Candidate Gender and Race for Democrats in Sample. AMCEs presented only for candidates who strongly support animal rights or have rescued farm animals.

References

Hainmueller, Jens, Dominik Hangartner and Teppei Yamamoto. 2015. “Validating vignette and conjoint survey experiments against real-world behavior.” *Proceedings of the National Academy of Sciences* 112(8):2395–2400.
